# Isolation of Biofilm-Forming Bacteria from Food Processing Equipment Surfaces and the Biofilm-Degrading Activity of *Bacillus licheniformis* YJE5

**DOI:** 10.3390/foods14213592

**Published:** 2025-10-22

**Authors:** Duna Yu, Jeong-Eun Lee, Min-Suk Rhee, Soon-Mi Shim, Ae-Son Om, Hary Yu, Moochang Kook

**Affiliations:** 1 Department of Biotechnology, Graduate School, Kyung Hee University, Yongin 17104, Republic of Korea; dbensk0205@khu.ac.kr; 2 Industry Academic Cooperation Foundation, Baewha Women’s University, Seoul 03039, Republic of Korea; ejungeunn@gmail.com; 3 Department of Biotechnology, College of Life Sciences and Biotechnology, Korea University, Seoul 02841, Republic of Korea; rheems@korea.ac.kr (M.-S.R.); bluet219@korea.ac.kr (H.Y.); 4 Department of Food Science and Biotechnology, Sejong University, Seoul 05006, Republic of Korea; soonmishim@sejong.ac.kr; 5 Department of Food and Nutrition, Hanyang University, Seoul 04763, Republic of Korea; aesonom@hanyang.ac.kr; 6 Department of Food and Nutrition, Baewha Women’s University, Seoul 03039, Republic of Korea

**Keywords:** food processing equipment surface, biofilm inhibition activity, *B. licheniformis* YJE5, intracellular substances

## Abstract

Food processing environments are prone to microbial contamination, where biofilm formation by persistent bacteria reduces hygiene and food safety. In this study, 27 biofilm-forming bacterial strains were isolated from processing equipment surfaces, with the dominant strains identified as *B. cereus* LE3, *B. cereus* YJBR3, and *P. cibi* F25. An environmental isolate, *B. licheniformis* YJE5, exhibited no hemolytic activity and demonstrated strong enzymatic potential. Intracellular substances (ICS) extracted from *B. licheniformis* YJE5, isolated from a food processing environment, significantly inhibited biofilm formation by *B. cereus* LE3, *B. cereus* YJBR3, and *P. cibi* F25 by 47–53% and degraded pre-formed biofilms by 37–44%. Light and confocal laser scanning microscopy confirmed pronounced structural degradation of biofilms following ICS treatment. API ZYM analysis further revealed multiple hydrolytic enzymes, indicating that enzymatic hydrolysis is central to biofilm degradation. Whole-genome sequencing showed a 4.3 Mbp chromosome with diverse metabolic pathways but no antibiotic resistance and virulence genes, confirming the strain’s safety. These findings suggest that *B. licheniformis* YJE5 represents a safe and eco-friendly candidate for controlling biofilm-associated contamination in food processing facilities.

## 1. Introduction

Environmental hygiene management in food processing environments is regarded as an important aspect of modern food safety programs to ensure the production of safe food [1]. The World Health Organization (WHO) reports that bacterial pathogens are responsible for approximately 60% of foodborne illnesses and 65% of foodborne deaths worldwide [2]. Working environments and equipment surfaces in food processing plants are frequently subject to contamination by spoilage or pathogenic microorganisms, which can serve as sources of cross-contamination, thereby reducing the effectiveness of food processing strategies and compromising both food quality and safety [3,4]. In particular, various stages of the food production chain, including raw material production, sorting, processing, packaging, and transportation, as well as exposure to pathogens in airborne aerosols, have been recognized as major causes of foodborne illness [5,6,7]. Cross-contamination of food with pathogenic bacteria in food processing equipment has been shown to occur primarily on conveyor belts and stainless-steel work surfaces [8,9,10]. According to a study by Lehto et al., which analyzed contamination levels from samples collected at six fresh vegetable processing plants in Finland between March and May 2009, total aerobic microbial counts exceeded 20 cfu/cm^2^ on all food-contact surfaces. Furthermore, 18% of non-mechanical food-contact surfaces showed moderate contamination levels, with an average of 2–10 cfu/cm^2^. The highest levels of total aerobic microbial contamination were found on centrifuge baskets used for washing lettuce leaves (100 cfu/cm^2^), cutter belts (90 cfu/cm^2^), and surfaces of floor-cleaning equipment (80 cfu/cm^2^). Even after cleaning, the lifting conveyor, floor, gloves, and peeling machine remained heavily contaminated, with total aerobic microbial counts ranging from 50 to 72 cfu/cm^2^ [11].

Ineffective or inadequate cleaning practices can leave food residues during food processing, which may promote bacterial attachment to equipment surfaces [12]. These bacteria can survive for several days on various surfaces, including food-contact materials and handling equipment. Some microorganisms produce extracellular polymeric substances (EPS), which help them adhere to each other and to surfaces. These substances form a protective layer, called a biofilm, within complex microbial communities, enabling the bacteria to survive and resist the effects of disinfectants [13,14].

Biofilms are communities of microorganisms that adhere firmly to solid surfaces and are embedded in a self-produced hydrated matrix of EPS, which contains polysaccharides, proteins, lipids, extracellular DNA (eDNA), and quorum-sensing molecules [15]. Bacteria that form biofilms are embedded in a substrate, which protects them from harsh environmental conditions such as extreme temperatures and pH, high salinity and pressure, and nutrient deprivation. They also exhibit high resistance to other external stressors, such as antibiotics, ultraviolet light, and chemical biocides [16,17,18]. Detergents used in cleaning processing equipment have limitations, such as residual toxicity, the development of resistant microorganisms, and environmental pollution. To address these issues, active research is needed on eco-friendly approaches that can reduce or replace the use of detergents, such as discovering antibacterial substances derived from microorganisms or utilizing antibacterial compounds of natural origin.

*Bacillus licheniformis*, a Gram-positive, endospore-forming, facultative anaerobic bacterium, is widely distributed as a saprophytic organism in the environment [19]. It is considered a generally recognized as safe (GRAS) organism by the U.S. Food and Drug Administration (FDA) [20]. *B. licheniformis* secretes a wide range of extracellular enzymes, including glycosidases and proteases, which can degrade cellulose, pectin, and keratin. It has been applied in agriculture, aquaculture, environmental protection, biomedicine, and food processing [21]. Compared with conventional enzyme-based strategies that rely primarily on extracellular DNases, proteases, and α-amylases to disrupt biofilms in food and industrial environments, this study investigated the biofilm-degrading activity of intracellular substances (ICS) from a newly isolated *B. licheniformis* strain recovered directly from a food processing environment. Although enzymatic degradation of biofilms by extracellular enzymes from *Bacillus* species has been reported, little is known about the biofilm-degrading potential and safety of ICS derived from *B. licheniformis* strains isolated from food processing environments. Accordingly, we isolated biofilm-forming bacteria from food processing equipment surfaces and evaluated the biofilm-inhibitory and biofilm-degrading activities of ICS from *B. licheniformis*. In addition to *Bacillus cereus* a well-known foodborne pathogen associated with spoilage and food intoxication, we evaluated activity against the recently reported, food-associated species *Proteus cibi*. Because some *Bacillus* strains used as probiotics may harbor antibiotic-resistance genes (ARGs) and virulence factors, necessitating genome-level vetting, we confirmed the biosafety of *B. licheniformis* by whole-genome analysis, which showed no ARGs and virulence genes. Collectively, these findings provide new insights for developing safe, environmentally friendly microbial agents to mitigate biofilm-related contamination in food processing facilities.

## 2. Materials and Methods

### 2.1. Sample Collection from Food Processing Facility

Between January and March 2024, a food processing facility located in Pyeongtaek, Gyeonggi-do, Republic of Korea (36.9941° N, 127.0889° E) was selected for sampling microorganisms from the surfaces of processing equipment. To assess microbial contamination, both surfaces directly and indirectly exposed to food were chosen. Each equipment surface was vigorously swabbed 10 times vertically, horizontally, and diagonally using a 3M Pipette Swab Plus kit (3M, St. Paul, MN, USA). Samples were collected after routine daily cleaning and disinfection of the facility. All swab samples were vortexed, and 1 mL of each was dispensed onto Plate Count Agar (PCA; Difco Laboratories, Detroit, MI, USA), which was then incubated at 37 °C for 48 h. To isolate airborne microorganisms in the food processing environment, PCA was placed on a vibration-free, flat surface and exposed to air for 30 min, followed by incubation at 37 °C for 48 h.

### 2.2. Isolation and Identification

A total of 3 mL of Tryptic Soy Broth (TSB; Difco Laboratories, Detroit, MI, USA) was dispensed into a 35 mm Petri dish (SPL Life Sciences Co., Ltd., Gyeonggi-do, Republic of Korea), and 1% (*v*/*v*) of the strain isolated from the surface of food processing equipment was inoculated and incubated at 37 °C for 48 h. After incubation, the biofilm formed on the bottom of the dish was washed three times with phosphate-buffered saline (PBS; Welgene Inc., Gyeongsan, Republic of Korea), and the dried biofilms were stained with 1 mL of 0.1% (*w*/*v*) crystal violet for 15 min. The stained dish was then washed three times with PBS, dried, and treated with 33% (*v*/*v*) acetic acid. Biofilm production was quantified by measuring absorbance at 595 nm using a microplate reader (BioTek, Winooski, VT, USA). Among the strains isolated from the surface of processing equipment, three strains that produced the strongest biofilms and one strain isolated from the food processing environment were identified by 16S rRNA sequencing (BIOFACT Co., Daejeon, Republic of Korea). The primers used for amplification were 27F (5′-AGAGTTTGATCMTGGCTCAG-3′) and 1492R (5′-TACGGTTACCTTGTTACGACTT-3′). The resulting sequences were compared with validated sequences of type strains using the 16S database on the EZBioCloud website (www.ezbiocloud.net) for identification. A phylogenetic tree was constructed using the neighbor-joining method in the MEGA-11 program.

### 2.3. Hemolysis Assay on Blood Agar

To determine the hemolytic activity of *B. licheniformis* YJE5 and biofilm-forming strains, blood agar plates were prepared by adding 5% (*v*/*v*) sheep blood (Kisan Bio Co., Seoul, Republic of Korea) to blood agar base (Kisan Bio Co., Seoul, Republic of Korea). The strains were streaked onto blood agar and incubated at 37 °C for 48 h. Hemolysis was assessed by the presence of clear zones surrounding colonies, following the method of Argyri et al. [22].

### 2.4. Preparation of Intracellular Substances from B. licheniformis YJE5

To prepare intracellular substances (ICS), 500 mL of de Man, Rogosa, and Sharpe (MRS) broth (Difco Laboratories, Detroit, MI, USA) was inoculated with 1% (*v*/*v*) *B. licheniformis* YJE5 and incubated at 37 °C for 24 h. After centrifugation at 10,000 rpm for 10 min, the supernatant was carefully discarded, and the cells were washed three times with PBS. The harvested cells were resuspended in 25 mL of PBS and sonicated (Vibra-cell, Sonics & Materials, Newtown, CT, USA) for 20 min at 20 kHz with a 10 s pulse and 35% amplitude. Following sonication, the lysate was centrifuged at 10,000 rpm for 10 min, and the supernatant was filtered through a 0.2 μm membrane filter for use in subsequent experiments [23].

### 2.5. BCA Protein Assay

Given that the biofilm-degrading activity of the ICS was hypothesized to arise primarily from peptides or enzymatic proteins, their overall concentration was quantified using the bicinchoninic acid (BCA) protein assay. This colorimetric method, which provides a proportional response to peptide bond content, was used to estimate the total amount of peptide- and protein-based components present in the crude extract. The assay was conducted using bovine serum albumin (BSA) as the calibration standard (Takara Bio, Inc., Tokyo, Japan), following the manufacturer’s protocol, and absorbance was measured at 562 nm with a microplate reader.

### 2.6. Biofilm Formation Inhibitory Activity

Each well of a 96-well plate (SPL Life Sciences Co., Ltd., Gyeonggi-do, Republic of Korea) was filled with 140 μL of TSB broth with 1% (*w*/*v*) dextrose and inoculated with 20 μL of 15 h cultures of *B. cereus* YJBR3, *B. cereus* LE3, and *P. cibi* F25. Subsequently, 20 μL of ICS was added, and the plates were incubated at 37 °C for 48 h. 20 µL of PBS was used as the negative control. After incubation, the supernatant was removed, and the biofilms formed at the bottom of the wells were washed three times with 200 μL of PBS and dried using a blow dryer. Biofilms were then stained with 100 μL of 0.1% (*w*/*v*) crystal violet for 15 min, washed three times with 200 μL of PBS, and dried. Finally, the stained biofilms were dissolved in 200 μL of 33% (*v*/*v*) acetic acid, and absorbance was measured at 595 nm using a microplate reader [24].

### 2.7. Biofilm Degradation Activity

Each well of a 96-well plate contained 160 μL of TSB broth with 1% (*w*/*v*) dextrose and was inoculated with 20 μL of 15 h cultures of *B. cereus* YJBR3, *B. cereus* LE3, and *P. cibi* F25, and then incubated at 37 °C for 48 h. The supernatant was then removed, and the biofilms formed at the bottom of the wells were washed three times with 200 μL of PBS and dried using a blow dryer. Subsequently, 50 μL of ICS was added, and the plates were incubated at 37 °C for 24 h. 50 µL of PBS was used as the negative control. The wells were again washed three times with 200 μL of PBS, dried, and stained for 15 min with 100 μL of 0.1% (*w*/*v*) crystal violet. After staining, the wells were washed three times with 200 μL of PBS, dried, and the biofilms were dissolved in 200 μL of 33% (*v*/*v*) acetic acid. Absorbance was measured at 595 nm using a microplate reader [25].

### 2.8. Observation of Biofilm Degradation by Light Microscopy

To visualize biofilms by light microscopy, 3 mL of TSB broth with 1% (*w*/*v*) dextrose were dispensed into 35 mm Petri dishes and inoculated with 1% (*v*/*v*) of 15 h cultures of *B. cereus* YJBR3, *B. cereus* LE3, and *P. cibi* F25. The plates were incubated at 37 °C for 24 h to allow biofilm formation. Following incubation, the attached biofilms were washed three times with PBS, dried, and treated with 1 mL of ICS, after which they were incubated at 37 °C for 24 h. 1 mL of PBS was used as the negative control. The supernatant was then removed, and the biofilms were washed three times with PBS, dried, and stained with 1 mL of 0.1% (*w*/*v*) crystal violet for 15 min. After staining, the plates were washed three times with PBS, dried, and the biofilms were observed by light microscopy (Dongwon CNS Edu, Seoul, Republic of Korea) [26].

### 2.9. Evaluation of Biofilm Formation in Response to NaCl and Dextrose Concentration

Biofilm formation at different NaCl and dextrose concentrations was assessed as follows. 3 mL of TSB broth containing 0.5–6% (*w*/*v*) NaCl or 0.25–5% (*w*/*v*) dextrose was dispensed into 35 mm Petri dishes and inoculated with 1% (*v*/*v*) cultures of *B. cereus* YJBR3 and *B. cereus* LE3. The plates were incubated at 37 °C for 48 h. After incubation, wells were washed three times with PBS, dried, and stained with 1 mL of 0.1% (*w*/*v*) crystal violet for 15 min. The supernatant was then removed, and the biofilms were washed three times with PBS, dried, and dissolved in 33% (*v*/*v*) acetic acid. Absorbance was measured at 595 nm using a microplate reader.

### 2.10. Confocal Laser Scanning Microscopy (CLSM) Imaging of the Biofilm

Biofilm degradation in response to ICS was assessed by CLSM with the LIVE/DEAD™ *Bac*Light™ Bacterial Viability Kit (L7012; Invitrogen, Carlsbad, CA, USA). Confocal dishes (SPL Life Sciences Co., Ltd., Gyeonggi-do, Republic of Korea) were inoculated with 1% (*v*/*v*) of the *B. cereus* LE3 in TSB broth with 0.5% (*w*/*v*) dextrose and 4% (*w*/*v*) NaCl, respectively, and incubated at 37 °C for 48 h. The attached biofilms were washed three times with PBS, dried, and treated with 1 mL each of ICS, α-amylase (Novozymes, Bagsværd, Denmark), and Alcalase^®^ 2.4 L FG (Novozymes, Bagsværd, Denmark), followed by incubation at 37 °C for 24 h. 1 mL of PBS was used as the negative control. After the supernatant was removed, the biofilms were washed three times with PBS to eliminate any unattached planktonic cells and dried. A mixture of the nucleic acid-binding dyes SYTO 9 and propidium iodide (PI) was prepared according to the manufacturer’s instructions and treated to the biofilms, and staining was carried out in the dark for 15 min. The stained biofilms were visualized using a Leica SP8-X CLSM (Leica Microsystems, Wetzlar, Germany), and 3D images were reconstructed with ImageJ software v1.54K [27,28].

### 2.11. Evaluation of α-Amylase and Protease Activities in B. licheniformis YJE5

Protease activity was evaluated by streaking onto a nutrient agar plate with 10% (*w*/*v*) skim milk (Difco Laboratories, Detroit, MI, USA) and incubating at 37 °C for 15 h, after which it was confirmed by the presence of a clear zone around the colonies [29]. α-amylase activity was evaluated by streaking onto a nutrient agar plate with 1% (*w*/*v*) starch and incubating at 37 °C for 15 h. After incubation, the plates were treated with iodine solution, and α-amylase activity was confirmed by the presence of a clear zone around the colonies [30].

### 2.12. Enzyme Activity Determination Using the API ZYM Kit

The enzyme activity of *B. licheniformis* YJE5 was evaluated using the API ZYM kit (BioMérieux, Marcy l’Etoile, France). 1 mL of MRS broth was inoculated at 1% (*v*/*v*) and incubated at 37 °C for 24 h. The culture was centrifuged at 10,000 rpm for 5 min and washed three times with PBS. The strain was then suspended in suspension medium (BioMérieux, Marcy l’Etoile, France) and adjusted to a turbidity corresponding to a 5–6 McFarland standard (BioMérieux, Marcy l’Etoile, France). An aliquot of 65 µL of the suspension was dispensed into each tube of the API ZYM kit, wrapped in aluminum foil to protect from light, and incubated at 37 °C for 4 h. After incubation, one drop each of ZYM A and ZYM B reagents (BioMérieux, Marcy l’Etoile, France) was added, and the mixture was allowed to react for 5 min. Enzyme activity was determined based on the reactions with 19 substrates, according to the reading table provided on the API website (http://apiweb.biomerieux.com).

### 2.13. Cell Viability Assay

Cell viability of ICS to RAW 264.7 cells (Korean Cell Line Bank, Seoul, Republic of Korea) was evaluated as follows. Cells (1 × 10^4^ cells/well) were seeded in 96-well plates and incubated at 37 °C, 5% CO_2_ for 24 h. After removing the supernatant, 180 µL of Dulbecco’s Modified Eagle Medium (DMEM; Cytiva, Marlborough, MA, USA) and 20 µL of the sample were added, and the cells were cultured for an additional 24 h. The supernatant was then removed, and the plates were washed three times with PBS. Subsequently, 180 µL of DMEM and 20 µL of MTT (Invitrogen, Carlsbad, CA, USA) solution (5 mg/mL) were added, and the cells were incubated for 4 h. After discarding the supernatant, the resulting formazan were dissolved by adding 100 µL of dimethyl sulfoxide (DMSO; Daejung, Siheung, Republic of Korea). The absorbance was measured at 570 nm on a microplate reader.

### 2.14. Genome Sequencing and Assembly

DNA extraction from *B. licheniformis* YJE5 was performed with the Mag-Bind^®^ Universal Pathogen Kit (Omega Bio-tek, Norcross, GA, USA) in accordance with the manufacturer’s protocol. A sequencing library was prepared with the SQK-LSK114 kit (Oxford Nanopore Technologies, Oxford, UK) and sequenced on a Nanopore Flongle flow cell. Base calling of the generated nanopore signals was performed using Guppy v6.5.7 [31] with the super-accuracy model and CUDA acceleration. Adapter sequences were trimmed from the reads using Porechop_ABI v0.5.0 [32], and the trimmed reads were assembled with FLYE v2.9.1 [33] using the nano-hq parameter. Assembly polishing was conducted in Medaka v1.8.0 (https://github.com/nanoporetech/medaka (accessed on 26 September 2024)) with the super-accuracy model to improve accuracy. The quality of the assembled genome was assessed with BUSCO v5.4.7 [34] against the micrococcales_odb10 database.

### 2.15. Prediction of Antimicrobial Resistance Gene and Potential Virulence Factors

The potential antibiotic resistance and virulence of *B. licheniformis* YJE5 were predicted. ResFinder v4.4.2 [35] and AMRFinderPlus v3.11.26 [36] were used to identify antibiotic resistance factors with default parameters. Potential virulence genes and toxin factors were analyzed against the VFDB [37] using DIAMOND v2.1.8 [38]. Toxin factors were filtered under relaxed conditions with an identity greater than 70% and query coverage greater than 60% [39].

### 2.16. Gene Prediction and Functional Annotation

Gene annotation and coding sequence prediction of *B. licheniformis* YJE5 were performed using the Prokka server. Prokka v1.14.5 [40] and Proksee v1.0.0a6 [41] were used for gene prediction, annotation, and construction of a circular map of the assembled genome. Clusters of Orthologous Groups (COG) and Gene Ontology (GO) annotation, as well as (Kyoto Encyclopedia of Genes and Genomes) KEGG pathway analysis of the assembled genome, were conducted using eggNOG-mapper v2.1.12, and the results were visualized.

### 2.17. Statistical Analysis

Data were analyzed using GraphPad Prism 8 (GraphPad Software, San Diego, CA, USA). Quantitative data are expressed as mean ± standard deviation. Statistical differences between two groups were evaluated using Student’s *t*-test. A *p*-value of <0.05 was considered statistically significant. Each experiment was conducted at least three times independently.

## 3. Results

### 3.1. Isolation and Identification of Bacteria from Food Processing Equipment Surfaces and Environments

**Figure 1:**
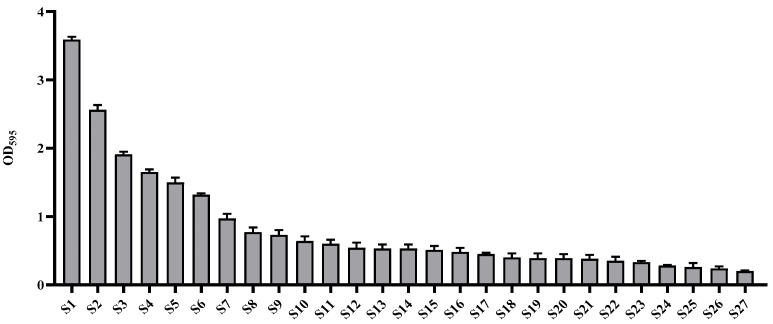
Evaluation of biofilm formation by wild-type strains isolated from food processing equipment surfaces.

**Figure 2:**
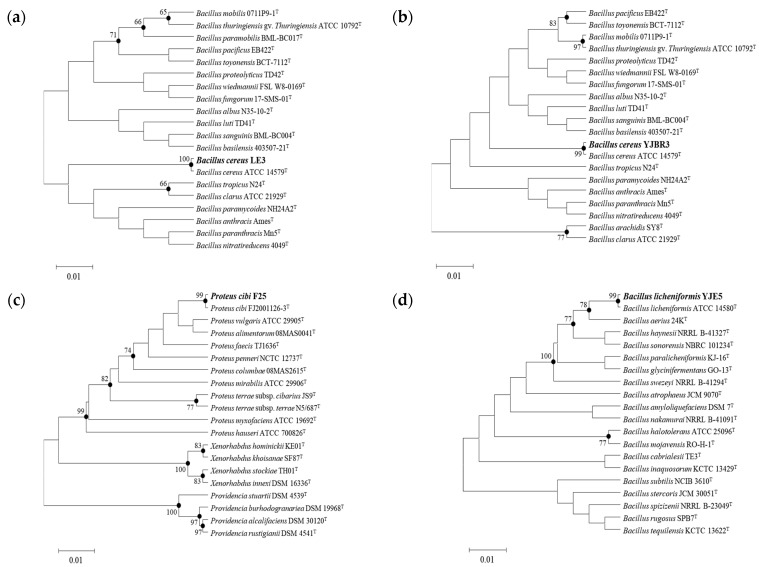
Neighbor-joining phylogenetic tree of the isolated strains. Bootstrap values (expressed as a percentage of 1000 replications) >65% are shown at the branch points. Bar, 0.01 substitutions per nucleotide position: (**a**) *B. cereus* LE3; (**b**) *B. cereus* YJBR3; (**c**) *P. cibi* F25; (**d**) *B. licheniformis* YJE5.

Among the strains isolated from food processing equipment surfaces, including cleaning brushes, food grinder blades, work tables, cutters, bubble washers, juicers, and food strainers, a total of 27 strains were found to form biofilms. Of these, the three strains (S1, S2, and S3) that exhibited the highest levels of biofilm formation, as determined by quantitative analysis using crystal violet staining (Figure 1), along with one strain isolated from the food processing environment, were subjected to 16S rRNA gene sequence analysis. The results showed that S1 (Figure 2a), isolated from a food grinder blade, exhibited 100% similarity to *Bacillus cereus* ATCC 14579^T^ and was identified as *Bacillus cereus* LE3. S2 (Figure 2b), isolated from a cleaning brush, showed 99.86% similarity to *Bacillus cereus* ATCC 14579^T^ and was identified as *Bacillus cereus* YJBR3. S3 (Figure 2c), isolated from a work table, showed 99.86% similarity to *Proteus cibi* FJ2001126-3^T^ and was identified as *Proteus cibi* F25. In addition, the strain isolated from the food processing environment showed 99.72% similarity to *Bacillus licheniformis* ATCC 14580^T^ and was identified as *Bacillus licheniformis* YJE5 (Figure 2d).

### 3.2. Hemolytic Activity

**Figure 3:**
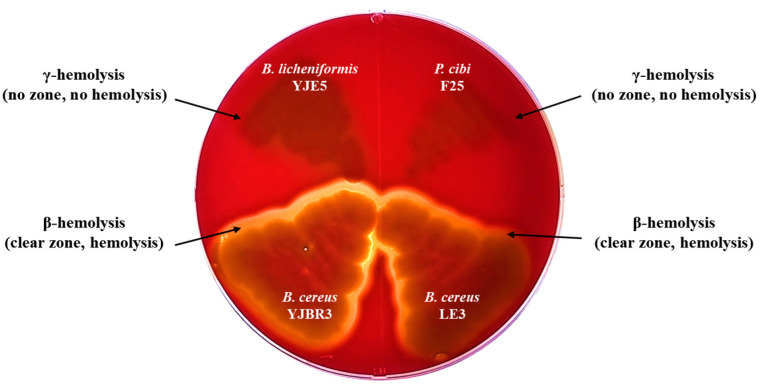
Hemolytic activity of *B. licheniformis* YJE5 and biofilm-forming strains.

Hemolysis is a phenomenon in which red blood cells are destroyed and hemoglobin is released. It can be classified into α-, β-, and γ-hemolysis based on the type of reaction observed on blood agar plates. α-Hemolysis refers to the partial lysis of red blood cells and is characterized by a green discoloration around the colonies. β-Hemolysis indicates the complete lysis of red blood cells, resulting in a clear zone surrounding the colonies. γ-Hemolysis refers to the absence of hemolysis, with no visible change around the colonies [42]. According to these criteria, *B*. *licheniformis* YJE5 did not exhibit hemolytic activity, whereas *B*. *cereus* YJBR3 and LE3 showed β-hemolysis. Additionally, *P*. *cibi* F25 did not induce hemolysis. *P. cibi* was first reported by Dai et al. (2019) [43], but detailed information on the hemolytic activity and biological properties of this species has not yet been confirmed (Figure 3).

### 3.3. Inhibition of Biofilm Formation by B. licheniformis YJE5 Intracellular Substances (ICS)

**Figure 4:**
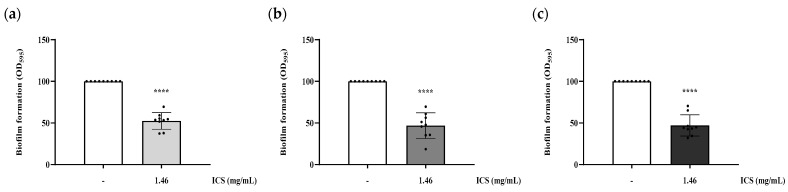
Inhibitory activity of ICS from *B. licheniformis* YJE5 against biofilm formation of (**a**) *B. cereus* YJBR3, (**b**) *B. cereus* LE3, and (**c**) *P. cibi* F25. Bars represent the mean ± SD (*n* = 9). “–” indicates untreated control, and “1.46” represents the concentration of ICS (mg/mL). Statistical analysis was performed using Student’s *t*-test; **** *p* < 0.0001 vs. the untreated control.

The inhibitory activity against biofilm formation was evaluated by co-incubating biofilm-forming bacteria with ICS. Treatment with 1.46 mg/mL ICS significantly reduced biofilm formation of *B. cereus* YJBR3, *B. cereus* LE3, and *P. cibi* F25 by 47.5%, 53.2%, and 52.97%, respectively, compared with the untreated control (*p* < 0.0001; Figure 4).

### 3.4. Biofilm-Degrading Activity of ICS from B. licheniformis YJE5

**Figure 5:**
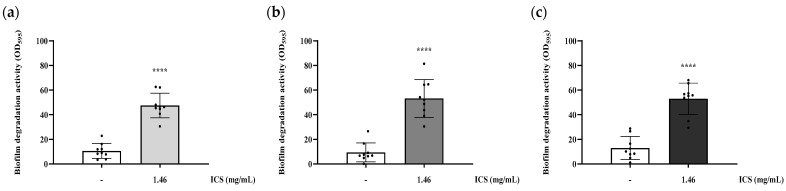
Biofilm-degrading activity of ICS from *B. licheniformis* YJE5 against pre-formed biofilms of (**a**) *B. cereus* YJBR3, (**b**) *B. cereus* LE3, and (**c**) *P. cibi* F25. Bars represent the mean ± SD (*n* = 9). “–” indicates untreated control, and “1.46” represents the concentration of ICS (mg/mL). Statistical analysis was performed using Student’s *t*-test; **** *p* < 0.0001 vs. the untreated control.

The biofilm-degrading activity was evaluated by treating pre-formed biofilms with ICS. Treatment with 1.46 mg/mL ICS significantly degraded pre-formed biofilms of *B. cereus* YJBR3, *B. cereus* LE3, and *P. cibi* F25 by 36.9%, 43.78%, and 40.01%, respectively, compared with the untreated control (*p* < 0.0001; Figure 5).

### 3.5. Observation of Biofilm Degradation Following Treatment with ICS from B. licheniformis YJE5

**Figure 6:**
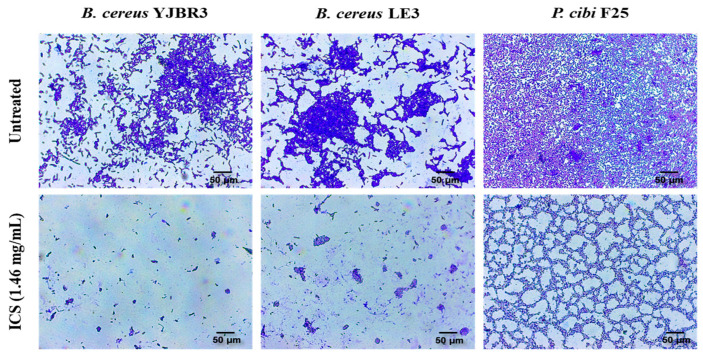
Light microscopy images of the biofilm-degrading effects of ICSs from *B. licheniformis* YJE5. The upper panels show untreated controls, while the lower panels depict biofilms treated with 1.46 mg/mL ICS. All images were captured at 40× magnification, and the scale bar represents 50 µm.

The biofilm-degrading activity of ICS was evaluated against pre-formed biofilms of *B. cereus* YJBR3, *B. cereus* LE3, and *P. cibi* F25. As shown in Figure 6, untreated control groups exhibited dense and compact biofilm structures. In contrast, biofilms treated with ICS displayed a marked reduction in density and structural integrity, with visibly dispersed and fragmented biofilm matrices.

### 3.6. Evaluation of Biofilm Formation at Various Concentrations of NaCl and Dextrose

**Figure 7:**
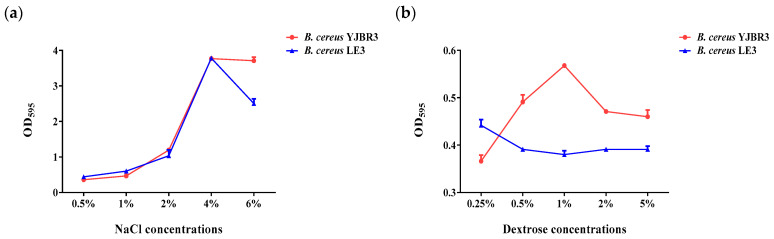
Evaluation of biofilm formation by *B. cereus* strains under conditions supplemented with various concentrations of NaCl and dextrose. (**a**) NaCl condition, (**b**) Dextrose condition. Values represent the mean ± SD (*n* = 3).

Salt and sugar residues remaining on the surfaces of food processing equipment can promote biofilm formation [44,45]. To simulate these conditions, this study evaluated the biofilm formation of *B. cereus*, a foodborne pathogen frequently associated with contamination in food processing environments, under various concentrations of NaCl and dextrose. As shown in Figure 7a, both *B. cereus* strains exhibited a concentration-dependent increase in biofilm formation with increasing NaCl concentrations, reaching a maximum at 4% NaCl. However, biofilm formation markedly decreased at 6% NaCl, indicating that excessive salinity exerts an inhibitory effect on biofilm development. As shown in Figure 7b, *B. cereus* YJBR3 exhibited the highest level of biofilm formation at 1% dextrose, after which biofilm formation declined with further increases in dextrose concentration. In contrast, *B. cereus* LE3 showed a moderate increase in biofilm formation beginning at 0.25% dextrose, followed by a gradual rise from 0.5% to 5%, although the overall response was less pronounced than that of *B. cereus* YJBR3. These findings indicate that moderate levels of salt and sugar residues can stimulate *B. cereus* biofilm formation, whereas excessive concentrations inhibit it, thereby highlighting the importance of controlling residual salt and sugar to mitigate biofilm-associated risks in food processing facilities.

### 3.7. CLSM Analysis of Biofilm-Degrading Activity of ICS from B. licheniformis YJE5

**Figure 8:**
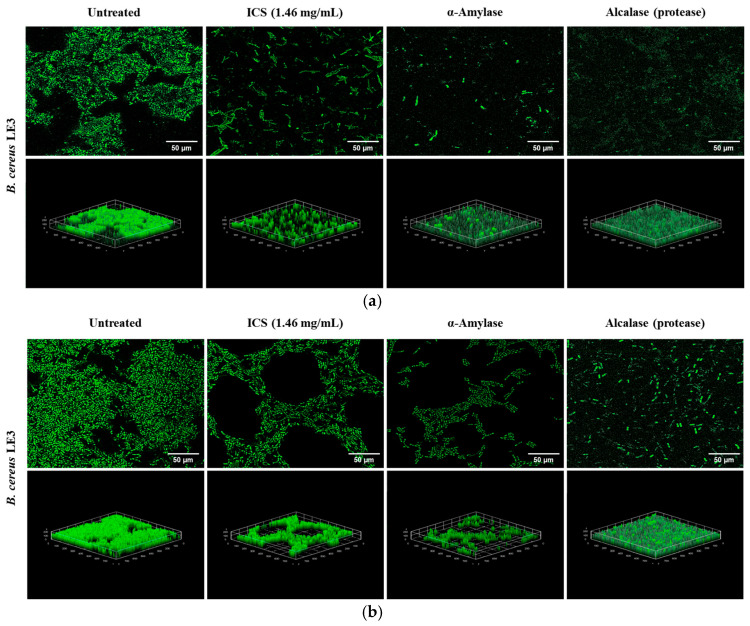
Confocal laser scanning microscopy images showing the degradation of mature biofilms formed in TSB broth supplemented with 4% NaCl and 0.5% dextrose by ICS from *B. licheniformis* YJE5: (**a**) 4% NaCl condition, (**b**) 0.5% dextrose condition. Mature biofilms were stained with SYTO9/propidium iodide. 2D images were captured at 100× magnification, and the scale bar represents 50 µm. 3D images were reconstructed with the ImageJ software v1.54K.

Confocal laser scanning microscopy was performed to assess the degradation of mature *B. cereus* LE3 biofilms formed under 4% NaCl (Figure 8a) and 0.5% dextrose (Figure 8b) conditions. Untreated controls exhibited dense and compact three-dimensional biofilm structures. In contrast, treatment with ICS (1.46 mg/mL) from *B. licheniformis* YJE5 significantly reduced the biofilm thickness and biomass. To further elucidate the mechanism of biofilm degradation by ICS, commercially available α-amylase and Alcalase (protease) were applied, both of which markedly disrupted the biofilm structure. *B. licheniformis* is a well-established industrial source of thermostable α-amylase [46] and extracellular subtilisin-type alkaline proteases, including subtilisin Carlsberg, which is marketed as Alcalase [47,48]. Previous studies have also demonstrated that sonication of *B. licheniformis* cells releases active intracellular enzymes into crude extracts [49], suggesting that amylolytic and proteolytic enzymes may be present in the ICS prepared in this work. Importantly, both glycoside hydrolases such as α-amylase and proteases are recognized as biofilm-disrupting enzymes capable of degrading extracellular polymeric substances (EPSs) and weakening biofilm structure [50]. Therefore, testing α-amylase and Alcalase provided mechanistic insight into the biofilm-degrading activity of ICS derived from *B. licheniformis* YJE5.

### 3.8. Enzymatic Activities of B. licheniformis YJE5

**Figure 9:**
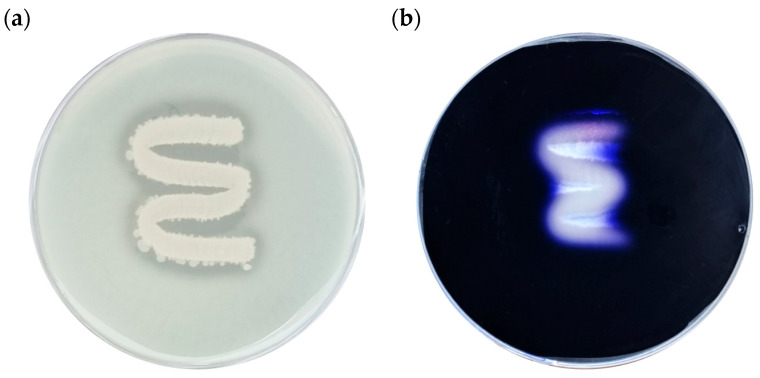
Evaluation of α-amylase and Alcalase (protease) activities of *B. licheniformis* YJE5: (**a**) Alcalase activity, (**b**) α-amylase activity.

As shown in Figure 9a, protease activity (Alcalase) was evident on skim milk agar, where a clear zone formed around the colonies, indicating casein degradation. Similarly, α-amylase activity was confirmed on starch agar (Figure 9b), where a distinct clear zone appeared following iodine staining, demonstrating hydrolysis of starch by amylolytic enzymes. These results verify that *B. licheniformis* YJE5 possesses both proteolytic and amylolytic activities, supporting its potential role in degrading protein and polysaccharide components of biofilm matrices.

### 3.9. Enzymatic Activity Profile of B. licheniformis YJE5 Determined Using the API ZYM Kit

The enzymatic activity profile of *B. licheniformis* YJE5 was determined using the API ZYM kit (Table 1). The strain exhibited strong activity for esterase (C4), cystine arylamidase, trypsin, α-chymotrypsin, acid phosphatase, and naphthol-AS-BI-phosphohydrolase. These findings indicate that enzymatic hydrolysis plays a central role in the biofilm-degrading activity of *B. licheniformis* YJE5, highlighting its potential as a biological agent for controlling biofilm-associated contamination in food processing environments.

### 3.10. Determination of Cell Viability of ICS from B. licheniformis YJE5

**Figure 10:**
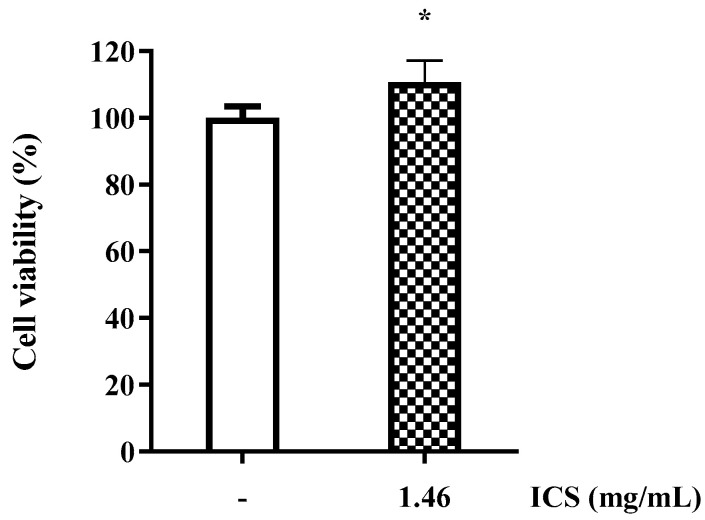
Cell viability of RAW 264.7 cells treated with ICS from *B. licheniformis* YJE5. Bars represent the mean ± SD (*n* = 6). “–” indicates untreated control, and “1.46” represents the concentration of ICS (mg/mL). Statistical analysis was performed using Student’s *t*-test; * *p* < 0.05 vs. the untreated control.

Cell viability was assessed using the MTT assay, which quantitatively measures survival based on the reduction of yellow MTT tetrazolium by mitochondrial dehydrogenases in living cells to form purple formazan crystals. The cytotoxicity of the ICS (1.46 mg/mL) derived from *B. licheniformis* YJE5 was evaluated, and cell viability exceeded 100% compared with the untreated control group (100% survival), confirming the absence of cytotoxicity (Figure 10).

### 3.11. Whole-Genome Sequencing Analysis of B. licheniformis YJE5

**Figure 11:**
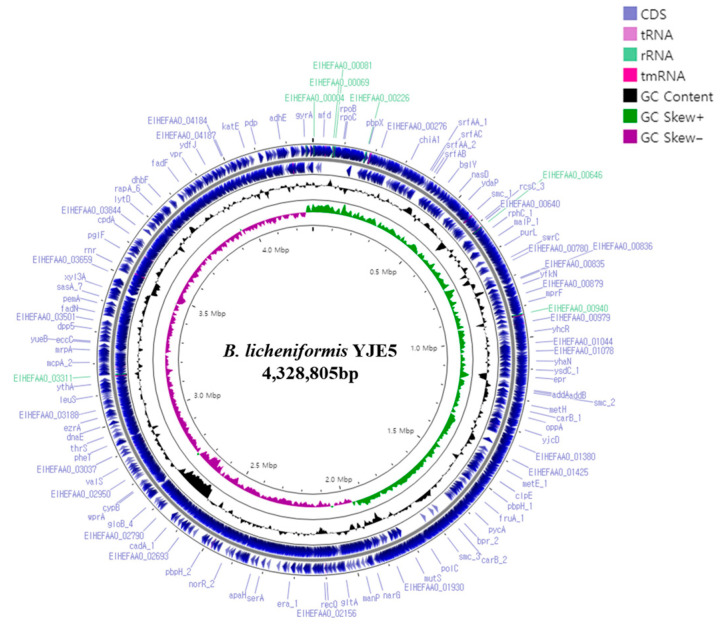
A circular genome map of *B. licheniformis* YJE5 generated using Prokka v1.14.5 and Proksee v1.0.0a6, illustrating the locations of coding sequences (CDSs) within the genome.

**Figure 12:**
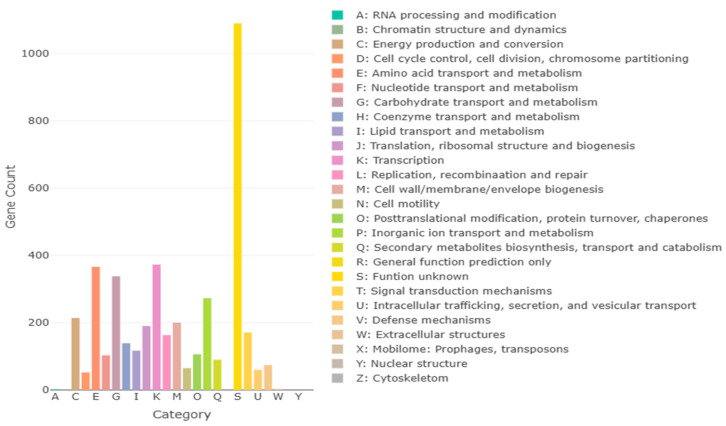
COG annotation results for *B. licheniformis* YJE5, with each letter representing a class of COG categories.

**Figure 13:**
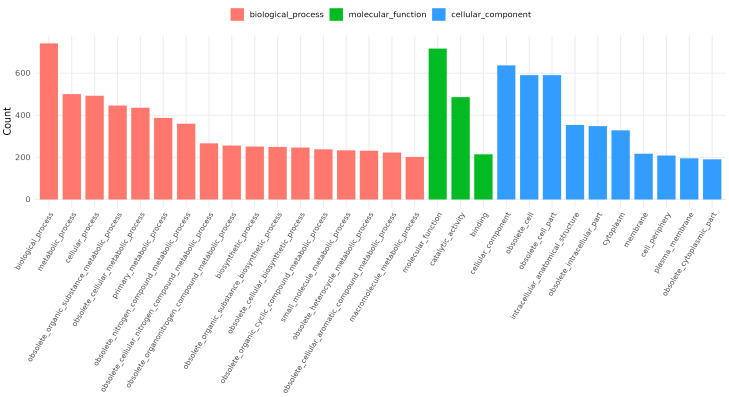
GO annotation results for *B. licheniformis* YJE5.

**Figure 14:**
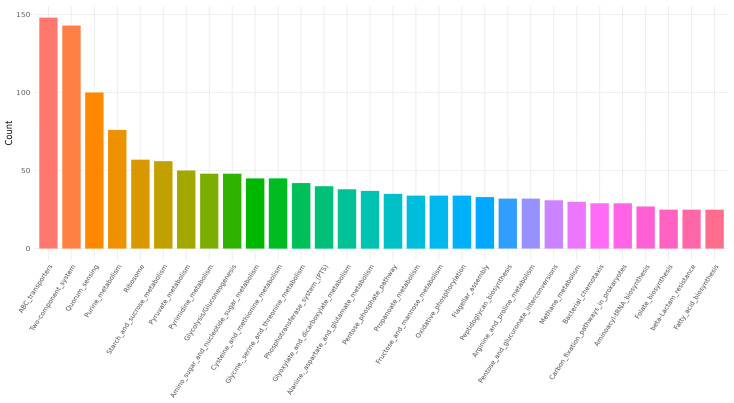
KEGG pathway results for *B. licheniformis* YJE5.

The complete genome of *B. licheniformis* YJE5 was successfully assembled into a single circular chromosome of 4,328,805 bp with a GC content of 45.95% (Figure 11, Table 2). Genome annotation identified 4391 coding sequences (CDSs), along with 24 rRNAs and 81 tRNAs. No plasmids were detected. Functional classification of genes using the COG database revealed that the majority of annotated genes were associated with general cellular functions, transcription, amino acid and carbohydrate metabolism, energy production, and transport mechanisms (Figure 12). GO categorization further highlighted that genes involved in biological processes, catalytic activity, and cellular components such as membranes and the cytoplasm were predominant, reflecting the organism’s capacity for versatile metabolic processes and environmental adaptation (Figure 13). KEGG pathway analysis identified a wide range of metabolic pathways, with notable enrichment in ABC transporters, two-component regulatory systems, quorum sensing, and central carbon metabolism (including purine, starch, and sucrose metabolism) (Figure 14). These results suggest that *B. licheniformis* YJE5 possesses robust nutrient utilization and regulatory networks, which may support its survival and biofilm-associated ecological functions. Importantly, screening with ResFinder and AMRFinderPlus detected no antibiotic resistance genes, and comparative analysis against the VFDB revealed no potential virulence or toxin factors in the genome. These findings underscore the strain’s potential safety for industrial and biotechnological applications.

## 4. Discussion

In this study, we isolated biofilm-forming bacterial strains from food processing environments and evaluated the biofilm-inhibiting and biofilm-degrading activities of *B. licheniformis* YJE5. Among the 27 biofilm-forming strains identified, the most dominant were *B. cereus* LE3, *B. cereus* YJBR3, and *P. cibi* F25, all of which demonstrated strong biofilm-forming capacities (Figure 1 and Figure 2). These results are consistent with earlier studies reporting the frequent isolation of *B. cereus* from food processing facilities, where it is known to adhere persistently to stainless steel and conveyor belt surfaces. Ryu and Beuchat further demonstrated that *B. cereus* biofilms are highly resistant to chlorine and other sanitizers, emphasizing the challenge of controlling this pathogen in food-contact environments [10]. Similarly, Lehto et al. observed microbial loads exceeding acceptable hygienic thresholds in fresh-cut vegetable plants, confirming the persistence of surface contamination despite routine cleaning [11]. These comparisons highlight the industrial relevance of our findings and reinforce the need for effective biofilm management strategies.

The hemolytic activity test revealed that *B. licheniformis* YJE5 was non-hemolytic, whereas *B. cereus* LE3 and YJBR3 exhibited *β*-hemolysis (Figure 3). This absence of hemolytic activity supports the safety profile of *B. licheniformis*, which has been recognized by the U.S. FDA as a generally regarded as safe (GRAS) organism. Similar findings were reported by Umanets et al., who emphasized the importance of genome-based safety assessments in probiotics, demonstrating the absence of hemolysis and virulence factors in well-characterized strains [39]. Together with our genomic data, which confirmed the lack of antimicrobial resistance or toxin genes, these results validate the potential of YJE5 as a safe candidate for industrial application.

The intracellular substances (ICS) extracted from *B. licheniformis* YJE5 demonstrated significant inhibitory effects on biofilm formation, with inhibition rates ranging from 47.5% to 53% against *B. cereus* YJBR3, *B. cereus* LE3, and *P. cibi* F25 (Figure 4). Moreover, ICS degraded mature biofilms with efficiencies of approximately 37–44% (Figure 5), and light microscopy confirmed reduced density and disruption of biofilm matrices (Figure 6). These results are consistent with previous studies showing that probiotic bacteria and enzymatic preparations can disrupt biofilm architecture. For example, Jaffar et al. demonstrated that *Lactobacillus* spp. degraded mature biofilms of *Aggregatibacter actinomycetemcomitans* [25], while Sun et al. reported that plant-derived protease ficin inhibited *Streptococcus mutans* biofilms [24]. The comparable activity observed in YJE5 ICS suggests that intracellular enzymes are key contributors to biofilm inhibition and degradation.

Environmental conditions also played a critical role in biofilm formation. Our results demonstrated that NaCl and dextrose concentrations modulated biofilm development in *B. cereus*, with certain concentrations enhancing while others suppressed biofilm formation (Figure 7). This is in agreement with Kwon et al., who reported that osmotic stress and nutrient availability strongly influence *B. cereus* biofilms in food processing environments [45]. Similarly, Rode et al. showed that stress conditions, including osmotic fluctuations, induced robust biofilm formation in *Staphylococcus aureus* [44]. Collectively, these findings highlight the potential risk posed by residual salt and sugar on food-contact surfaces, underscoring the importance of stringent cleaning practices to mitigate biofilm-associated contamination.

Mechanistic insights into the biofilm-degrading activity of YJE5 were obtained through enzyme assays and confocal microscopy. Both *α*-amylase and protease (Alcalase) activities were confirmed (Figure 9 and Table 1), and CLSM imaging revealed that treatment with either ICS or commercial *α*-amylase and Alcalase disrupted biofilm matrices formed under NaCl and dextrose supplementation (Figure 8). These results are consistent with earlier reports indicating that glycoside hydrolases and proteases are potent biofilm-disrupting enzymes due to their ability to degrade extracellular polymeric substances (EPS) [47]. Alcalase, a subtilisin-type protease from *B. licheniformis*, is widely utilized in industrial applications [47], while Pawar and Rathod showed that intracellular protease production in *B. licheniformis* can be enhanced through sonication, similar to our preparation of ICS [23]. Thus, the enzymatic composition of YJE5 ICS likely underpins its biofilm-degrading activity, reinforcing the potential application of this strain in eco-friendly biofilm control. In food processing facilities, disinfectants such as hypochlorous acid water often fail to completely remove mature biofilms. If ICS can weaken or partially degrade biofilms, subsequent disinfection becomes more effective. In this regard, ICS should be considered a complementary agent rather than a replacement for disinfectants. Given that sampling in this study was performed after routine daily cleaning and disinfection, ICS could be applied as a pre-treatment or post-wash step to loosen established deposits, used periodically as a clean-in-place (CIP) additive for “biofilm-prone sites,” or applied for targeted remediation on equipment with recurrent biofilm accumulation. These findings directly demonstrate the potential of YJE5-derived ICS as an adjunctive treatment for persistent biofilm contamination in food processing environments.

In recent years, biologically based biofilm-inhibition strategies utilizing microorganisms have gained increasing attention as sustainable and safe alternatives to chemical disinfectants. Probiotic or enzymatically active bacterial strains can produce antimicrobial metabolites, compete for adhesion sites, or secrete biofilm-disrupting enzymes, thereby suppressing the growth of pathogenic biofilms. For instance, probiotic *Lactobacillus* biofilms have been reported to inactivate *Listeria monocytogenes*, reducing planktonic cells by 0.66–2.01 log and biofilm cells by 0.40–1.69 log CFU cm^2^ [51]. Similarly, several lactic acid bacteria and *Bacillus*-based biocontrol strains have demonstrated biofilm-inhibitory or degrading activities against foodborne pathogens such as *Staphylococcus aureus* and *Salmonella enterica* through the secretion of biosurfactants and hydrolytic enzymes [3,50]. Collectively, these findings highlight the potential of biologically derived agents, such as ICS, to serve as eco-friendly and effective tools for controlling biofilm-associated contamination in food processing environments.

Finally, whole-genome sequencing of *B. licheniformis* YJE5 provided comprehensive genomic evidence of safety and functional capacity. The genome consisted of a 4.3 Mbp circular chromosome with diverse coding sequences related to carbohydrate metabolism, proteolysis, and stress adaptation (Figure 11, Figure 12, Figure 13 and Figure 14 and Table 2). Importantly, no virulence or resistance genes were detected, in line with previous reports describing *B. licheniformis* as a safe hydrolase-producing strain with probiotic potential [21]. Functional enrichment in KEGG pathways such as quorum sensing and ABC transporters further suggests that YJE5 possesses regulatory and adaptive traits conducive to survival in competitive environments, while maintaining biosafety for industrial application.

Our study demonstrates that *B. licheniformis* YJE5 combines enzymatic biofilm-degrading capacity with a robust safety profile, positioning it as a promising microbial resource for food safety management. By comparing our results with previous research, it is evident that YJE5 not only aligns with established findings on the enzymatic degradation of biofilms but also contributes novel insights into the role of intracellular enzyme extracts in targeting persistent pathogens such as *B. cereus* and *P. cibi*. These findings provide a strong foundation for the development of sustainable, biological strategies to mitigate biofilm contamination in food processing environments.

## 5. Conclusions

Intracellular substances (ICS) extracted from *B. licheniformis* YJE5, isolated from a food processing environment, exhibited notable inhibitory and degradative effects against biofilms formed by *B. cereus* and *P. cibi*. Microscopic observations confirmed significant biofilm disruption, while genome analysis verified the absence of antibiotic resistance and virulence genes, confirming strain safety. Although partial, the biofilm-inhibitory effect is practically meaningful, as ICS can complement conventional disinfectants by weakening biofilm matrices. These findings highlight *B. licheniformis* YJE5 as an eco-friendly and biosafe resource for improving hygiene in food processing facilities.

## Figures and Tables

**Table 1:** Enzyme activity of *B. licheniformis* YJE5 using API ZYM kit.

Enzymes	YJE5	Enzymes	YJE5
Control	- ^(1)^	Acid phosphatase	+
Alkaline phosphatase	w ^(2)^	Naphthol-AS-BI-phosphohydrolase	+
Esterase (C4)	+ ^(3)^	*α*-galactosidase	-
Esterase Lipase (C8)	w	*β*-glucuronidase	-
Lipase (C14)	-	*β*-glucosidase	-
Leucine arylamidase	w	*α*-glucosidase	-
Valine arylamidase	w	*β*-glucosidase	-
Crystine arylamidase	+	N-acetyl-*β*-glucosaminidase	-
Trypsin	+	*α*-mannosidase	-
*α*-chymotrypsin	+	*α*-fucosidase	-

^(1)^ -; negative, ^(2)^ w; weak positive, ^(3)^ +; positive.

**Table 2:** Genome features of *B. licheniformis* YJE5.

Features	
Genome size (bp)	4,328,805
GC content (%)	45.95
Coding sequence (CDS)	4391
rRNA	24
tRNA	81

## Data Availability

The original contributions presented in this study are included in the article. Further inquiries can be directed to the corresponding author. The assembled and annotated genome of *Bacillus licheniformis* YJE5 has been deposited in the NCBI BioProject database under the accession number PRJNA1345241. The 16S rRNA gene sequences of *Bacillus cereus* YJBR3, *Bacillus cereus* LE3, and *Proteus cibi* F25 have been deposited in the NCBI GenBank database under the accession numbers PX458902, PX458925, and PX458928, respectively.
